# Respiratory Symptoms and Pulmonary Function Testes in Lead Exposed Workers

**DOI:** 10.5812/ircmj.4134

**Published:** 2012-11-15

**Authors:** Mohammad Reza Khazdair, Mohammad Hossein Boskabady, Reza Afshari, Bita Dadpour, Amir Behforouz, Mohammad Javidi, Abbasali Abbasnezhad, Valiallah Moradi, Seyed Saleh Tabatabaie

**Affiliations:** 1 Department of Physiology, School of Medicine and pharmaceutical Research Center, Mashhad University of Medical Sciences, Mashhad, IR Iran; 2 Department of Toxicology, School of Medicine, Mashhad University of Medical Sciences, Mashhad, IR Iran; 3 Toxicology Laboratory, Imam Reza Hospital, Mashhad, IR Iran; 4 Iranian Red Crescent Hospital, Dubai, UAE

**Keywords:** Lead, Signs and Symptoms, Respiratory, Respiratory Function Tests

## Abstract

**Background:**

The exposure to noxious agwents such as lead my cause lung disorders.

**Objectives:**

In the present study, pulmonary function tests and self-reported respiratory symptoms in lead exposure workers were compared with matched control subjects.

**Materials and Methods:**

The frequency of respiratory symptoms were evaluated in a sample of 108 lead exposure workers and 100 control subjects with similar age using a questionnaire including questions on respiratory symptoms in the past year. Pulmonary function tests (PFT) were also measured in lead exposure workers and in controls.

**Results:**

Most lead exposure workers (63%) reported work-related respiratory symptoms. Chest tightness (26%), cough (17%) and sputum (16%) were the most common symptoms and only 6% of lead exposure workers reported wheezing (P < 0.001 for all case except wheezing). Most PFT values were also significantly reduced among lead exposure workers (P < 0.05 to P < 0.001 except MEF75, MEF50, MEF25, and MMEF. The lead concentration in urine and serum of lead exposure workers were significantly higher than control (P < 0.001 for both cases).

**Conclusions:**

These results showed that c lead exposure workers have higher frequencies of respiratory symptoms higher serum and urine lead concentration but lower PFT values.

## 1. Background

In spite of effort to limit for use of lead components industry services, this heavy metal remains as one of the industrial environmental pollution (1-3). Although the effect environmental pollution on morphology and respiratory physiology is demonstrated the effects of environmental lead on respiratory system is controversial and more studies needed to clarify this effect (4, 5). It is suggested that lead pollution may contribute in pathogenesis of pulmonary cancers, asthma, COPD however, there is not confident result in this regard yet (6-8). Epidemiological studies suggests that lead may play a role in causing asthma as the incidence of asthma in workers exposed to lead as well as children that live in high levels of lead pollution places is increased (9, 10). In addition these studies showed significant relationship between blood lead concentration and IgE (9, 10).

Increase of IgE and some inflammatory cytokines in serum of laboratory models and children exposed to lead as well as release of inflammatory mediator from the cells and macrophages exposed to lead in a cell culture model were reported. However, some reports indicated no change or even decrease of serum immunoglobins in laboratory models exposer lead (11-16). Therefore more studies are need for evaluation of immunotoxic effect of lead exposure and the role of this metal in incidence and severity of asthma and other respiratory diseases. In addition in experimental studies on animal models exposed to lead respiratory system morphologic changes as well as increased tracheal responsiveness was observed which could be demonstrate the role of lead in pathogenesis pathology of asthma disease but definite document regarding the role of lead in pathology of asthma or other respiratory diseases is not available yet (17).

## 2. Objectives

Therefore, in the present study pulmonary function tests and self-reported respiratory symptoms in lead exposed workers of a car battery manufactory were compared with matched control subjects.

## 3. Materials and Methods

### 3.1. Subjects

One hundred eight lead exposed workers of a car battery manufactory (mean age ± SD; 31.31 ± 6.91, 102 male and 6 female) and 100 control subjects (mean age ± SD; 34.13 ± 7.81, 92 male and 8 female) were studied. The control subjects were chosen from visitors to the Medical Center with approximately the same age as lead exposed workers. All lead exposed workers employed in a car battery manufactory for 1 to 13 years (mean employment duration ± SD; 4.65 ± 2.99 years). All workers were worked 8 hours in a day, 6 day in each week. All studied subjects were non-smokers; none reported a recent respiratory infection or gave a history of asthma or COPD prior to lead exposure. The study was approved by the Ethical Committee of the institution of Mashhad University of Medical Sceinces, and each subject gave informed consent.

### 3.2. Protocol

A Farsi questionnaire was used to assess the prevalence and severity of respiratory (wheezing, tightness, cough and sputum), (Table 1). Questionnaire on respiratory symptoms was designed in accordance with several previous questionnaires of similar studies by expert groups (18-23). Common risk factors like smoking habit, atopy and history of allergic reactions were also asked. Moreover, the participants answered questions regarding all employment period (working hours per day and total working period).

**Table 1:** The criteria for respiratory symptom severity score

Symptom and Frequency	Score
**Wheezing**	
**None**	0
**During mild exercise (walking)**	1
**During heavy exercise**	2
**At rest**	3
**Cough**	
**None**	0
**During mild exercise (walking)**	1
**During heavy exercise**	2
**At rest**	3
**Tightness**	
**None**	0
**During mild exercise (walking)**	1
**During heavy exercise**	2
**At rest**	3
**Sputum**	
**None**	0
**Small volumes of non-purulent sputum**	1
**Large volumes of non-purulent sputum**	2
**Purulent sputum**	3
**Total score**	12

Pulmonary function tests in lead exposed workers and control subjects were measured using a spirometer with a pneumotachograph sensor (Model ST90, Fukuda, Sangyo Co., Ltd. Japan). Prior to pulmonary function testing, the PFT measurement technique was demonstrated by the operator, and subjects were encouraged and supervised throughout test performance. Pulmonary function testing was performed using the acceptability standards outlined by the American Thoracic Society (ATS) with subjects in a standing position and wearing nose clips (24). Measurements of PFT values were carried out between 1000 and 1700 hours. Pulmonary function tests were performed three times in each subject. The highest level for forced vital capacity (FVC), forced expiratory volume in one second (FEV1), peak expiratory flow (PEF), maximal mid expiratory flow (MMEF) and maximal expiratory flow at 75%, 50%, and 25% of the FVC (MEF75, MEF50, and MEF25 respectively) were taken independently from the three measurements.

### 3.3. Measurement of Serum and Urine Lead Concentration

#### 3.3.1. Serum Sampling

By venepuncture 3-4 ml of whole blood were drawn into a plastic syringe. A1ml sample was placed in a plastic tube containing K3EDTA and stored at 4°C for subsequent determination of whole blood lead. The remainder of the sample, placed in another Tube also containing K3EDTA, was immediately centrifuged at 800rev/min for 15 min; the plasma obtained was stored at-20°C for subsequent lead determination.

#### 3.3.2. Urine Sampling

About 50 ml of urine sample was transferred into a metal-free plastic bottle immediately after collection in a clinical paper cup, and then acidified by the addition of 200 µ1 nitric acid. The urine samples were kept frozen until analysis.

#### 3.3.3. Atomic Absorption Spectrometry (AAS)

Sample is dissolved in an acid mixture. The resulting solutions were analyzed by Atomic Absorption Spectrometry. Standards solutions with known concentrations of the elements were used to establish calibration data. Calibration was verified through analysis of NIST or NIST traceable certified reference materials. All determinations were performed using a Perkin Elmer atomic absorption spectrophotometer Model 3030 (made in US) with a lead hollow cathode lamp (HCL) and (EDL) was used, equipped with a HGA 400 graphite furnace and a deuterium background corrector. Nitrogen was used as purge gas for the graphite furnace.

### 3.4. Data Analysis

The data of age, PFT values, and lead concentration were expressed as mean ± SD and data of respiratory and allergic symptoms as percentage of each group having the corresponding symptom. Differences in the data of symptoms between lead exposed workers and control group were tested by Chi-Squared analysis (2X2 contingency tables). Differences in the data of symptom between lead exposed workers and control group and also between rest and working periods were tested by calculating Relative Risk and the 95% Confidence Intervals (RR, 95% CI.). The data of PFT values and serum concentration between lead exposed workers and control groups were tested using unpaired t tests. The correlation between PFT values and respiratory symptoms with urine and serum lead concentration was performed using least square test. A two-sided p value of 0.05 was the criterion for statistical significance. All analyses were performed with SPSS software (version 11.5, SPSS Inc. USA).

## 4. Results

### 4.1. Comparison of Respiratory and Allergic Symptoms Between Lead Exposed Workers and Control Group

**Figure 1:**
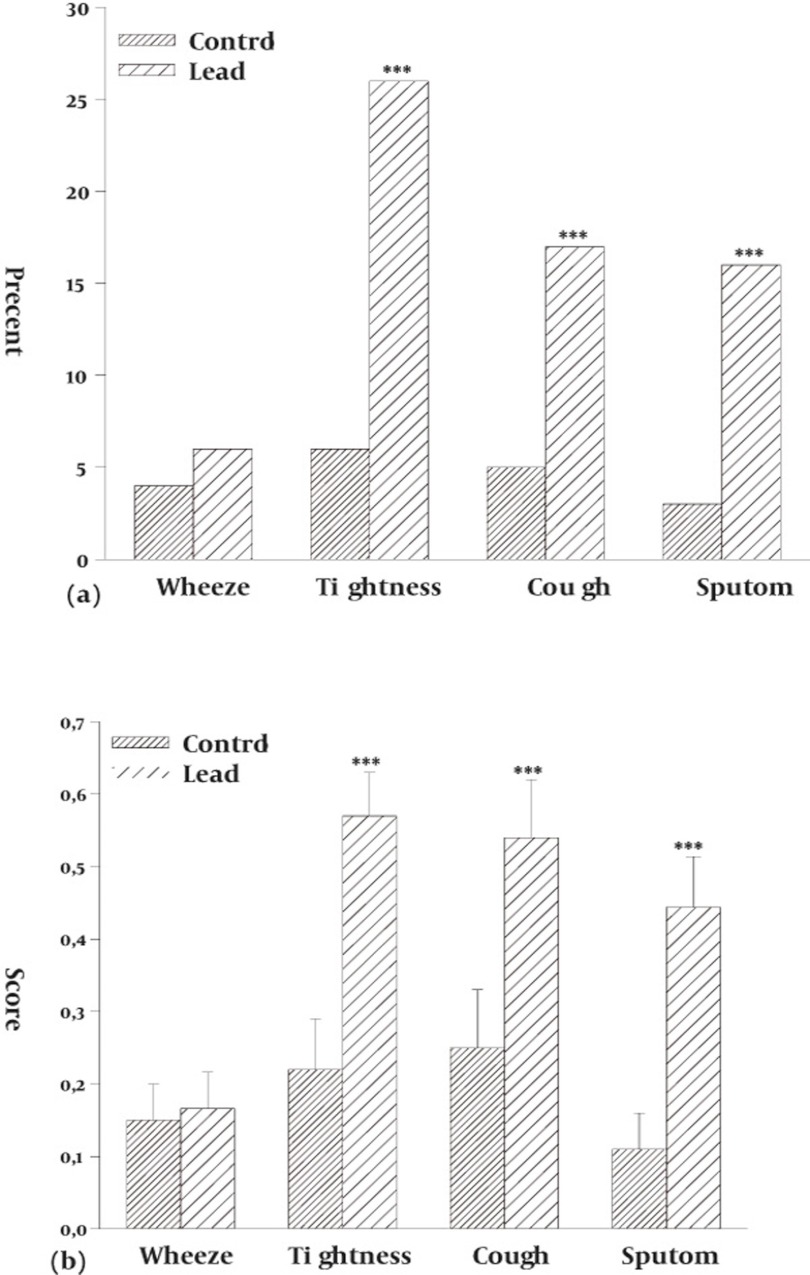
Comparison of prevalence (a) and severity (b) of respiratory symptoms between control subjects and lead exposed workers (for Control group, n = 100 and lead exposed group, n=108). ***: P < 0.001

Most lead exposure workers (63%) reported work-related respiratory symptoms. Sputum (16%), cough (17%), wheezing (6%) and Tightness of breathe (26%). The prevalence of sputum, cough and tightness were higher in lead exposed workers compared to control group (P < 0.001 for all case) but there was no significant difference in the prevalence of wheezing between two groups (Figure 1a). The severities of all respiratory symptoms were also higher in lead exposed workers compared to control group for sputum, cough and tightness (P < 0.001 for all case, Figure 1b)

### 4.2. Comparison of PFT Values Between Lead Exposed Workers and Control Group

**Figure 2:**
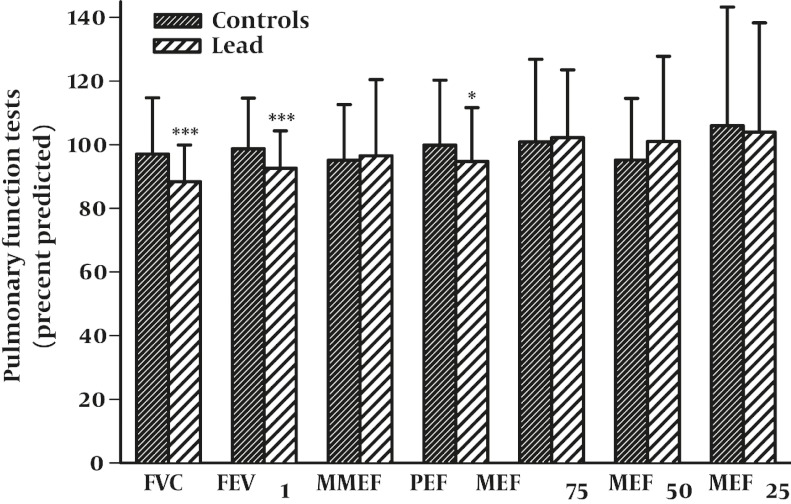
Comparison of pulmonary functional tests (PFT) between control subjects and lead exposedworkers (for Control group, n = 100 and lead exposed group, n = 108). Values were presents as mean ± SD. FVC: forced vital capacity, FEV1: forced expiratory volume in one second, MMEF: maximal mid expiratory flow, PEF: peak expiratory flow, MEF75, MEF50, and MEF25: maximal expiratory flow at 75%, 50%, and 25% of the FVC, respectively. Statistical significance between two groups; *: P < 0.05, ***: P < 0.001

The values of FVC (forced vital capacity), FEV1 (forced expiratory volume) and PEF (peak expiratory flow) were lower in lead exposed workers than control subjects (P < 0.05 to P < 0.001, Figure 2). However, there was no significant difference between MMEF, MEF75, MEF50, and MEF25 between two groups (Figure 2).

### 4.3. Comparison of Serum and Urine Lead Concentration Between Lead Exposed Workers and Control Group

The concentration of urine and serum lead in lead exposed workers were higher than those of control subjects (P < 0.0001, Table 2).

**Table 2:** Comparison of urine lead and serum lead between control subjects and lead exposed workers

Parameter	Control	Lead exposed Workers	Stat. dif.
**Urine lead**	23.560±1.63	76.84±5.76	P < 0.0001
**Serum lead**	72.51±3.46	370.50±22.182	P < 0.0001

### 4.4. Correlation Between PFT Values and Respiratory Symptoms With Urine and Serum Lead Concentration in Lead Exposed Workers

The serum lead concentration were correlated with all PFT values except FEV1 (P < 0.05 to P < 0.01), but PFT values were correlated with urine lead concentration for only MMEF and MEF25 (P < 0.05 for all cases, Table 3).

**Table 3:** Correlation between PFT values with urine (Urin L) and serum (Serum L) lead concentration in lead exposed workers

PFT Values	FVC		FEV1		MMEF		PEF		MEF75		MEF50		MEF25	
	r	p	R	P	r	p	r	P	r	p	r	p	r	p
**Urine L**	0.12	0.31	0.02	0.88	0.22	0.05	0.14	0.20	0.12	0.29	0.19	0.09	0.24	0.03
**Serum L**	0.21	0.05	0.02	0.85	0.28	0.01	0.21	0.05	0.22	0.05	0.30	0.01	0.25	0.02

## 5. Discussion

The results of the present study showed significant greater respiratory symptoms compared to control subjects. The severities of respiratory symptoms also were significantly higher among lead exposed workers than controls. In addition PFT values in lead exposed worker were lower compared to control group. The urine and serum lead concentration in lead exposed workers was significantly higher than control subjects. There was also significant correlation between serum lead concentration and PFT values. These results indicate the effect of the exposures to lead on respiratory status of these workers and confirms that exposure to lead in work environment induces respiratory disorder.

The increased urine and serum lead concentration in lead exposed workers was supported by the a previous observation indicating severe smoke inhalation of closed-space fires of a tertiary burn center was associated with a more than 2-fold increase in blood lead levels of lead (25). The results of the present study showed high prevalence and severity of Tightness, sputum and cough among lead exposed workers but lower prevalence of wheeze which are more compatible with COPD rather than asthma. These findings are also supported by the results of a previous study showing higher prevalence of respiratory symptoms for phlegm, shortness of breath and diagnosed asthma in industrial workers exposed with lead (6). However, there was no increased likelihood of asthma diagnosis or symptoms among young children with lead poisoning (26). The possible reason for this discrepancy is perhaps the long period of lead exposure in studied workers in the present study. In addition, an elevated incidence of lung cancer was observed in smelter workers exposed to lead (7).

However, the findings of our study more clearly indicate the effect of exposure to lead on respiratory symptoms as well as PFT values. There was also significant correlation between serum lead concentration and change of most PFT values. These observations confirm that exposure to lead in environment may responsible for development of respiratory symptoms. In addition reduction of PFT values and existence of respiratory symptoms in lead exposed workers indicate a permanent respiratory change in these workers.

The results of our study showed a considerable reduction in some values of PFT in lead exposed workers compared to control subject. In fact, it was shown that blood lead concentration was associated with increased bronchial hyperresponsivenes (27). In addition, the concentrations of lead, especially over a long period, may cause irreversible morphological alterations in the, rat lung tissue (15). The reason of less obvious reduction in MMEF and MEF75 MEF50, and MEF25 values in lead exposed workers in the present study may indicate the higher effect of lead exposure on larger airways (28). The reason of the greater effect of lead exposure on larger airways may be due to particle size of this heavy metal in the factory environment. The higher effect of lead exposure on FVC, FEV1 and PEF may also indicate that lead exposure may induce COPD like changes in the lung. This conclusion also supported by lower effect of lead exposure on prevalence and severity of wheeze and its higher effect on chest tightness, cough and sputum. The increased blood lead levels of battery and exhaust workers and reduction of their PFT was shown previously which support the results of the present study (29).

In conclusion, the results of this study showed that lead exposure in work environment can cause a higher frequency of respiratory symptoms. PFT values were also significantly reduced among lead exposed workers compared to control subjects indicating a permanent change of respiratory system may occur due to lead exposure. Therefore, the results suggest a relationship of environmental lead exposure and respiratory status.
